# The Performance Implications of Job Insecurity: The Sequential Mediating Effect of Job Stress and Organizational Commitment, and the Buffering Role of Ethical Leadership

**DOI:** 10.3390/ijerph17217837

**Published:** 2020-10-26

**Authors:** Min-Jik Kim, Byung-Jik Kim

**Affiliations:** 1 Institute of Finance and Banking, Seoul National University, 1 Kwanak-ro, Seoul 08826, Korea; kmj8113@snu.ac.kr; 2 College of Business Administration, University of Ulsan, Ulsan 44610, Korea

**Keywords:** job insecurity, organizational performance, job stress, organizational commitment, ethical leadership, moderated sequential mediation model

## Abstract

Although previous works have examined how job insecurity affects the perceptions, attitudes, and behaviors of members in an organization, those studies have not paid enough attention to the relationship between job insecurity and performance or the mediating processes in that relationship. Considering that organizational performance is a fundamental target or purpose, investigating it is greatly needed. This research examines both mediating factors and a moderator in the link between job insecurity and organizational performance by building a moderated sequential mediation model. To be specific, we hypothesize that the degree of an employee’s job stress and organizational commitment sequentially mediate the relationship between job insecurity and performance. Furthermore, ethical leadership could moderate the association between job insecurity and job stress. Using a three-wave data set gathered from 301 currently working employees in South Korea, we reveal that not only do job stress and organizational commitment sequentially mediate the job insecurity–performance link, but also that ethical leadership plays a buffering role of in the job insecurity–job stress link. Our findings suggest that the degree of job stress and organizational commitment (as mediators), as well as ethical leadership (as a moderator), function as intermediating mechanisms in the job insecurity–performance link.

## 1. Introduction

Rapid environmental changes, such as the COVID-19 pandemic, are likely to function as an extreme shock and lead to economic crises and recession. Therefore, many firms have undertaken large-scale restructuring and downsizing, causing employees to feel a sense of job insecurity [1,2], which is a perception of uncertainty about the continuity of employment [3,4]. Extensive previous works have revealed that job insecurity critically influences various employee and organizational outcomes by functioning as a serious job stressor. For instance, job insecurity significantly predicts a low level of mental/physical health and work attitudes/behavior among organizational members (e.g., satisfaction at work, organizational commitment, job involvement, work engagement, creativity, and extra-role behavior), as well as predicting a low level of both individual and organizational performance [3,5,6,7,8,9,10,11,12,13,14,15,16,17,18,19].

Although previous works on job insecurity have investigated its influence on important organizational outcomes, we still see some research gaps in this area [17]. First, previous studies on the job insecurity–organizational outcomes link have not fully examined the effects of job insecurity on organizational performance [12,17]. Instead, they revealed that job insecurity critically affects the attitudes/behaviors and physical/mental health of individual organizational members. However, organizational performance is a critical target when the goal is to survive in a competitive business environment. Also, the quality or effectiveness of employee-level outcomes (e.g., attitudes, behaviors, and physical and mental health) should ultimately be evaluated in terms of the level of organizational performance. In other words, the association between job insecurity and organizational performance has been relatively ignored, despite its importance to an organization’s long-term survival. Therefore, studies about the job insecurity–performance link are greatly needed.

Furthermore, extant research on the relationship between job insecurity and performance have concentrated mainly on individual-level performance [16,17]. For instance, Staufenbiel and König [20] used self-rating and supervisor-rating performance scales to reveal the influence of job insecurity on individual employee performance. Wang et al. [19] also examined the association between job insecurity and individual-level performance by using supervisor-rating performance measures. Clearly, the individual performances of organizational members are a crucial factor in organizational performance. But we here concentrate on how job insecurity affects organizational-level performance [12,17].

Second, as some scholars who delved into the association between job insecurity and performance have already pointed out [5,10,17,19], the link between them has an inconclusive direction. For example, some meta-analyses demonstrated that job insecurity tends to decrease individual-level performance [5,10], which suggests that job insecurity plays a detrimental role by boosting member stress and generating negative perceptions of social exchanges with the firm [6,7,11,12,15,16,17,21]. Other papers have reported that job insecurity is unrelated to performance [18,20,22], and still other scholars have found that, from a job-preservation motivation perspective, unstable work security can increase members’ work-related motivation [20,23]. Those inconclusive associations could be due to a lack of work on the mediators and moderators that affect this link [17].

Third, previous studies on job insecurity have mostly ignored the importance of leadership [17]. Although the literature contains reports about various contingent factors that moderate the effects of job insecurity, including self-esteem about work-related achievements, emotional intelligence, organizational communication, organizational justice, social support from supervisors, job control, job self-efficacy, and economic vulnerability [19,22,24,25,26,27,28,29,30,31], little research has investigated the moderating role of leadership. Given that leadership has substantial effects on employees by building their attitudes and behaviors [32] and that leaders often symbolize an organization itself [33], examining the contextual role of leadership in the job insecurity–organizational performance link is important.

To fill that research gap, we here delve into the underlying mechanisms that affect the job insecurity–performance link. To be specific, we suggest that employee job stress and organizational commitment could sequentially mediate that link. Also, ethical leadership could moderate the influence that job insecurity has on the job stress of employees. Job stress is defined as a member’s negative emotional reactions, such as fear, anxiety, anger, and sadness, toward job stressors [34]. Many previous studies have reported that job insecurity in employees functions as a stressor that increases the degree of job stress [28,35,36,37,38]. Existing research also reveals that employee job stress diminishes the degree of organizational commitment [39,40,41,42,43].

Organizational commitment is an employee’s psychological attachment and willingness to be devoted to an organization [44]. It is a pivotal concept because organizationally committed employees are likely to demonstrate a high level of satisfaction at work, extra-role behavior, in-role/out-role performance, and organizational performance and a low level of turnover [44,45,46,47,48,49]. Existing works that rely on social exchange theory [50] have found that members who experience a great deal of job stress seek to hold a balance between themselves and their organization by decreasing the level of organizational commitment [39,40,50,51]. Therefore, we hypothesized that a decreased level of organizational commitment caused by job stress would diminish the level of organizational performance [45,48,52].

More important, our results suggest that ethical leadership could be a critical contextual factor that moderates the relationship between job insecurity and job stress. Among the various contextual features in an organization, we focus on the role of leadership because of its importance in an organization [32,33].

To be specific, we propose that ethical (i.e., fair and just) leadership could function as a buffering factor that decreases the increase in job stress produced by job insecurity [53,54,55,56]. Ethical leadership can be defined as “the demonstration of normatively appropriate conduct through personal actions and interpersonal relationships, and the promotion of such conduct to followers through two-way communication, reinforcement, and decision-making” (Brown, Trevino, & Harrison, 2005, p. 120). When the degree of ethical leadership is high, the increase in job stress caused by job insecurity is likely to be alleviated. In other words, even when employees are in danger of losing their job at the firm, they might not feel extreme stress if they are confident that the company makes decisions fairly and ethically (ethical leadership) [53,54,55,56,57]. In contrast, employees who feel that their leader is unethical might perceive that their organization evaluates them unfairly. Eventually, employees become unable to justify and accept their feelings of instability because of unfairness in the decision making.

In summary, in this paper, we examine the influence of job insecurity on organizational performance, focusing on the mechanisms that underlie that link. We hypothesize that job insecurity will decrease the level of organizational performance through the sequential mediation of job stress and organizational commitment. Furthermore, ethical leadership could moderate the relationship between job insecurity and job stress. Thus, this research contributes to the job insecurity literature in the following ways. First, we investigate how job insecurity affects organizational performance. Second, we attempt to resolve the inconclusive association between job insecurity and performance by investigating two mediators and a moderator. Third, we examine the critical role of leadership to explain the influence of job insecurity.

## 2. Theory and Hypotheses

### 2.1. Job Insecurity and Job Stress

Many existing works on job insecurity have reported that job insecurity substantially increases the level of employee job stress. Job insecurity functions as a critical and serious stressor by both decreasing a job’s controllability and predictability and increasing an employee’s perceptions of threat and loss [28,35,36,37,38].

Job insecurity, by definition, involves a great deal of uncertainty about whether organizational members can stably maintain their jobs. In that situation, employees cannot predict and control the possibility of keeping their employment. As a result of job insecurity, employees might doubt their abilities to get basic resources that are critical to well-being, such as a social identity, wage, social connectedness, and social status [17,27,58].

Furthermore, job insecurity directly deteriorates the level of satisfaction of basic psychological needs, satisfaction including autonomy, competence, and relatedness [59,60,61,62]. Based on the feeling of job insecurity, employees are likely to perceive that they do not have enough abilities to deal with their work competently, feeling that they may be rejected by their colleagues and organization due to the lack of capability. Moreover, they would feel anxiety and worry, because they feel that they are worthless and unworthy in the organization. Those negative feelings have been known to function as a direct job stressor [35,37].

**Hypothesis** **1.** Job insecurity is positively associated with employee job stress.

### 2.2. Job Stress and Organizational Commitment

Many scholars have shown that employee job stress is closely related to employee attitudes in an organization, including organizational commitment [39,40,41,42,43]. Organizational commitment indicates the extent of an employee’s psychological attachment and intention to contribute to the organization [46,52,63]. Employees who are highly committed to their organization tend to accept the goals of their organization as their own goals, pursuing profit maximization for the organization beyond their self-interest [64,65], which eventually produces positive outcomes such as a high level of satisfaction, extra-role behavior, and in-role performance at work [45,46,52].

The social exchange perspective provides theoretical supports for the relationship between job stress and organizational commitment. Based on social exchange theory [50], we expect the quality of the exchange between employees and their organization to determine the level of organizational commitment [51]. According to that theory, members who experience a great deal of job stress seek to hold a balance between themselves and their organization, sometimes by trying to give back the negative experience of job stress [50,51]. Given the fundamental power disparity between an organization and its members, employees might not be able to directly return the uncomfortable feelings that originate as job stress. Instead, they might passively and indirectly retaliate against the organization by reducing their positive attitudes toward it (e.g., organizational commitment). Decreasing their level of organizational commitment can allow employees to recover a feeling of psychological balance [50,51].

**Hypothesis** **2.** Job stress is negatively related to organizational commitment.

### 2.3. Organizational Commitment and Organizational Performance

We also argue that a higher level of organizational commitment ultimately contributes to organizational performance. Organizational commitment has been shown to be critical in organizations because of its close relationship with outcomes such as satisfaction at work, turnover, in-role/extra-role performance, and organizational performance [44,45,46,47,48,49]. Meta-analyses have reported that organizational commitment is closely associated with performance, which is a fundamental organizational goal [47,48,49].

Employees with a high degree of organizational commitment can perceive and consider the aims, values, and interests of their organization as their own. Therefore, they are likely to make sincere efforts to fulfill the expectations of the organization by working hard. Those efforts are expressed as enhanced in-role/extra-role performance, along with high-quality attitudes and behaviors [44,66,67]. Thus, enhanced organizational commitment is likely to be shared among employees in an organization through a social-contagion process [68]. Shared organizational commitment can be summarized at the collective level as a “common attitudinal ground” [69].

If collective organizational commitment is created through social interactions in an organization, members might feel that they want to be loyal to the organization and invest their cognitive, emotional, and physical resources to realize the organization’s goals [70]. In that situation, employees tend to help their colleagues achieve those aims by providing an increased level of cooperative behavior that boosts the quality of interactions involved in conducting work-related tasks, facilitating morale and cohesiveness in an organization. Eventually, those enhanced attitudes and behaviors directly increase organizational effectiveness and performance [71,72].

**Hypothesis** **3.** Organizational commitment is positively associated with organizational performance.

### 2.4. Sequential Mediating Role of Job Stress and Organizational Commitment between Job Insecurity and Organizational Performance

Given those arguments and hypotheses, we propose that job stress and organizational commitment could function as sequential mediators in the job insecurity–performance link. To be specific, job insecurity can decrease organizational performance by increasing the degree of job stress, which diminishes the degree of organizational commitment.

**Hypothesis** **4.** Job stress and organizational commitment function as sequential mediators in the job insecurity–organizational performance link.

### 2.5. Moderation Effect of Ethical Leadership in the Job Insecurity–Job Stress Link

As described above, it is clear that job insecurity increases the level of employee job stress. However, we believe that theoretical and empirical associations are not always established in real organizations because many contextual factors can moderate the job insecurity–job stress link. Specifically, we propose that ethical leadership could function as a buffering factor that decreases the negative effects that job insecurity has on job stress [56].

Members in an organization tend to anthropomorphize the organization (Dowling, 2001), i.e., they consider the organization to be a virtual and invisible person [73,74]. In other words, members tend to ascribe human-like characteristics (e.g., motives and intentions) to their organization. Moreover, members can regard the leader as a symbolic actor who represents the organization as a whole, eventually perceiving the behaviors of the leader to be the behaviors of the organization [33]. In that situation, employees often determine whether they trust the organization based on whether they trust their leaders. Among the various leadership behaviors that form an organization’s norms and standards, we here concentrate on ethical behaviors by leaders [53,75], proposing that ethical leadership functions as an important moderator of the job insecurity–job stress link [56].

We propose that when the level of ethical leadership is high, the increase that job insecurity causes in job stress could be alleviated [56]. Even though members are in danger of losing their jobs at the company, they might not feel extreme stress if they perceive that the company has ethical leadership. In that situation, employees might recognize that they will be treated ethically, which makes them more likely to understand and even accept job instability [53,54,55,56]. No matter how seriously the employees experience job insecurity, if they believe they are being evaluated in a fair and ethical manner, their level of job stress could decrease substantially. A moral leader can thus positively influence how employees perceive job insecurity by allowing them to participate in ethical leadership as a moral person [56]. Conversely, when members perceive that the company evaluates unfairly them through an unethical leader, they could decide that the job insecurity they are experiencing cannot be justified [53,54,55,56]. That would prevent them from understanding or accepting their feeling of job insecurity, eventually creating more severe job stress. In other words, ethical leadership functions as a critical contingent factor that moderates the job insecurity–job stress link.

**Hypothesis** **5.** Ethical leadership would moderate the job insecurity–job stress link.

**Figure 1:**
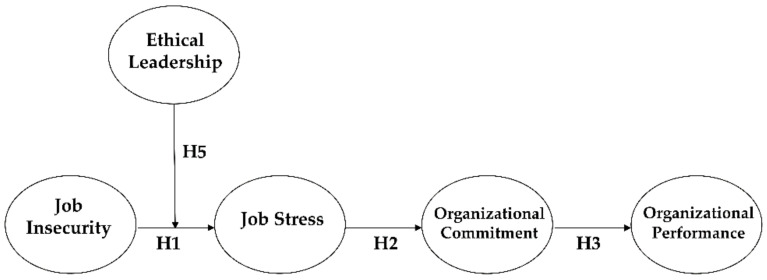
Theoretical Model.

Taken our hypotheses together, we suggest that job insecurity can enhance organizational performance through the sequential mediating effects of job stress and organizational commitment. Moreover, ethical leadership moderates the association between job insecurity and job stress (Please See Figure 1).

## 3. Method

### 3.1. Samples and Procedure

Using an online survey system, South Korean workers participated in our survey during three distinct time points. Our data were collected before the COVID-19 pandemic. Therefore, unfortunately, we cannot reflect the influence of the pandemic. To decrease the harmful effects of sampling bias, we conducted random sampling. To resolve the limitations embedded in cross-sectional data, we gathered distinct data with from employees and human resource management directors at 3 distinct time points.

At Time one, 512 workers responded to the survey; at Time two, 378 workers participated; and at Time three, 335 workers participated. The intervals between time points were 4 or 5 weeks. Our survey system was opened for 2 or 3 days each at each time point to provide enough time for the respondents. When the system was open, they could access it whenever they tried. After we closed the system each time, we eliminated incomplete and missing data from the raw dataset. In the end, we received usable data from 301 employees. The rate of response was 58.79%. The features of the respondents are described in Table 1.

### 3.2. Measures

We measured our variables using a 5-point Likert scale ranging from 1 to 5 (strongly disagree to strongly agree). By gathering data from distinct origins, we attempted to diminish the potential harms of common method bias.

#### 3.2.1. Job Insecurity (Time Point 1, Collected from Employees)

This paper utilized 4 items of the job insecurity scale [76]. The items were as follows: “If my current organization were facing economic problems, my job would be the first to go,” “I will not be able to keep my present job as long as I wish,” “My job is not a secure one,” and “My job will not be there although I want it” (Cronbach’s alpha = 0.88).

#### 3.2.2. Ethical Leadership (Time Point 1, Collected from Employees)

Ethical leadership was assessed by the seven items of the ethical leadership scale by Brown and his colleagues [75]. In this paper, we utilized these items, including “My leader disciplines employees who violate ethical standards,” “My leader conducts his/her personal life in an ethical manner,” “My leader discusses business ethics or values with employees,” “My leader sets an example of how to do things the right way in terms of ethics,” “When making decisions, my leader asks what is the right thing to do,” “My leader listens to what employees have to say,” and “My supervisor can be trusted” (Cronbach’s alpha = 0.91).

#### 3.2.3. Job Stress (Time Point 2, Gathered from Employees)

To evaluate the degree of job stress, 4 items of the job stress scale that were adapted from the scales of previous studies [77,78] were used. Items were as follows: “At work, I felt stressed during the last 30 days,” “At work, I felt anxious during the last 30 days,” “At work, I felt frustrated during the last 30 days,” and “At work I felt angry during the last 30 days” (Cronbach’s alpha = 0.89).

#### 3.2.4. Organizational Commitment (Time Point 2, Collected from Employees)

At time point 2, to measure organizational commitment, we used four items of the organizational commitment scale of Meyer and Allen [52]. Items were as follows: “I really feel as if my organization’s problems are my own,” “I feel a strong sense of belonging to my organization,” “I would be very happy to spend the rest of my career working with my organization,” and “I feel emotionally attached to my organization” (Cronbach’s alpha = 0.90).

#### 3.2.5. Organizational Performance (Time Point 3, Gathered from Directors of the Human Resource Management Department)

Directors of the human resource management department at each firm evaluated the degree of organizational performance using 4 items (Cronbach’s alpha = 0.92). Items were taken from the previous research [12,79] and consisted of “Our firm is more efficient than our competitors,” “Our overall management performance is superior to our competitors,” “Our financial performance is excellent in comparison to our competitors,” “Our firm is more productive than our competitors.” Through gathering data from distinct origins, this paper attempted to diminish the potential harms of common method bias.

To be specific, the organizational performance was evaluated by a concept of “perceived organizational performance” based on the following logic: previous works in the organizational behavior sphere had reported that subjective perceptions or assessments of specific phenomena by members of an organization (e.g., the employee’s perception of the level of his or her organizational performance) are likely to reflect the phenomenon itself (e.g., the actual degree of organizational performance) as precisely as objective scales (e.g., operating profit or net profit for measuring organizational performance). This argument is originated in the fact that the perceptions of members of an organization may substantially create the realities in an organization [12,57]. By relying on the above logic, we suggest that using the subjective scale may be worthful although we acknowledge the strengths of objective scales as well as the weakness of subjective measures.

#### 3.2.6. Control Variables (Time Point 2)

The organizational performance was controlled by firm size and the industry type of each firm, and organizational commitment was controlled by employee’s working experience (in months), gender, job title, and educational level [80,81].

### 3.3. Statistical Analysis

The associations between the variables were evaluated using a Pearson correlation analysis. As suggested by Anderson and Gerbing [82], we took a two-step approach that included the measurement and the structural model. This paper implemented a confirmatory factor analysis (CFA) to test the validity of the measurement model. After that, we conducted a structural equation modeling (SEM) analysis by building a moderated sequential mediation model to test our structural model. We used the maximum likelihood (ML) estimator to perform the SEM. Moreover, to evaluate whether the mediation effect is statistically significant, we used a bootstrapping method by using the 95% bias-corrected confidence interval (CI) to evaluate the mean indirect effect. If the CI did not include zero, the indirect effect was interpreted to be statistically significant at the 0.05 level.

To check if the fit indices are acceptable, we used a variety of fit indices, including the comparative fit index (CFI), the Tucker–Lewis index (TLI), and the root mean square error of approximation (RMSEA). Multiple scholars have reported that CFI and TLI greater than 0.90 and RMSEA less than 0.06 are good [83]. Furthermore, we conducted a bootstrapping analysis to check the significance of the indirect effect [84].

## 4. Results

### 4.1. Basic Statistics

The results of the correlation analysis are presented in Table 2. The study variables including job insecurity, ethical leadership, organizational commitment, and organizational performance were significantly associated.

### 4.2. Measurement Model

We conducted a CFA for all items to check the goodness-of-fit of the measurement model. Because our four psychometric constructs from the same employee (i.e., job insecurity, job stress, organizational commitment, and ethical leadership) are incorporated in our model, the discriminant validity of the three variables was checked. The 4-factor model showed a good fit to the data (χ^2^ (df = 140) = 237.758; CFI = 0.974; TLI = 0.969; RMSEA= 0.048). Additionally, we performed a series of chi-square difference tests by consequently comparing the 4-factor model to a 3-factor (χ^2^ (df = 143) = 788.651; CFI = 0.830; TLI = 0.797; RMSEA= 0.123), 2-factor (χ^2^ (df = 145) = 1216.979; CFI = 0.718; TLI = 0.667; RMSEA = 0.157), and one-factor (χ^2^ (df = 146) = 1771.215; CFI = 0.572; TLI = 0.499; RMSEA= 0.193) models. The results of the chi-square difference tests demonstrated that the 4-factor model was better than other alternative models. Therefore, we suggest that the four variables have an adequate level of discriminant validity.

### 4.3. Structural Model

In this paper, we made a moderated sequential mediation model that contains both mediating and moderating structures between job insecurity and performance. In the mediating structure, the association between job insecurity and performance was sequentially mediated by job stress and organizational commitment. In the moderating structure, ethical leadership moderates the influence of job insecurity on job stress.

To check the severity of multicollinearity bias in job insecurity and ethical leadership, this research computed the variance inflation factors (VIF) and tolerances [85]. The VIF values for job insecurity and ethical leadership were 1.008 and 1.028, and the tolerance values were 0.992 and 0.973, respectively. Considering the criterion for the values [85], this paper concludes that job insecurity and ethical leadership are relatively free from the problem of multi-collinearity.

#### 4.3.1. Analysis for Mediating Effect

**Figure 2:**
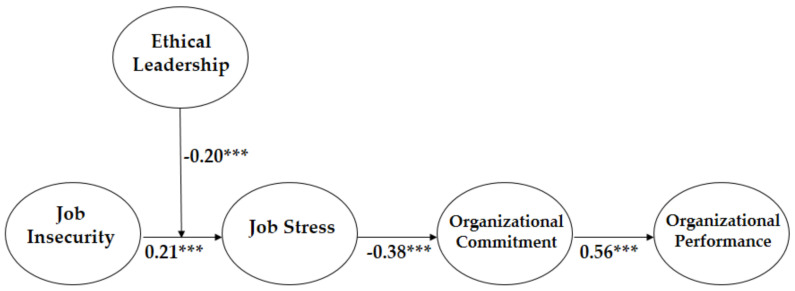
The Result of Coefficient Values of our Research Model. *** *p* < 0.001.

We performed SEM analysis to test our research model. The fit indices of the model were sufficient to accept (χ^2^ = 338.324 (df = 220), CFI = 0.968, TLI = 0.959, and RMSEA = 0.042). The control variables including gender, job title, education, and firm size are not significant, only excluding working experience. Including the control factors, the final model demonstrated that job insecurity increases job stress (β = 0.21, *p* < 0.001), thus supporting Hypothesis 1, that job stress decreases commitment of members (β = −0.38, *p* < 0.001), thus supporting Hypothesis 2, and that organizational commitment increases organizational performance (β = 0.56, *p* < 0.001), thus supporting Hypothesis 3 (Please See Figure 2).

#### 4.3.2. Analysis for Moderating Effect

To test whether ethical leadership moderates the job insecurity–job stress link, we made an interaction term by conducting a mean-centering procedure. Centered variables have been known to have various strong points, including minimizing the loss of correlations and the possibility of multi-collinearity [85].

**Figure 3:**
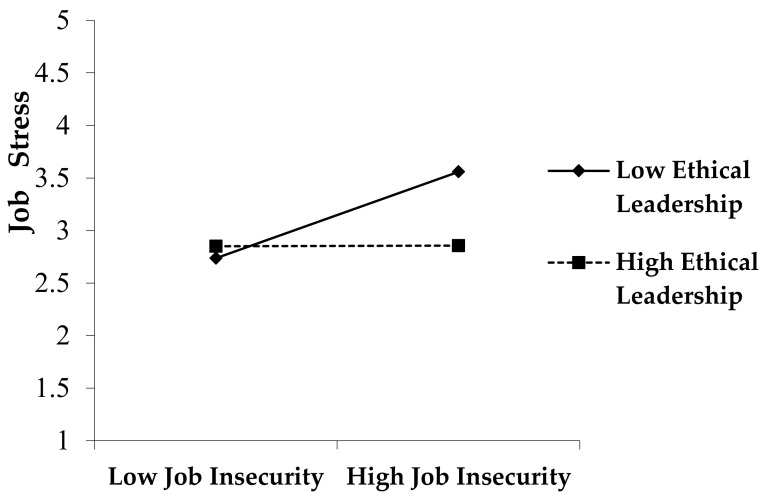
Moderating Effect of Ethical Leadership in the Job insecurity–Job Stress link.

The interaction term (β = −0.20, *p* < 0.001) was statistically significant, indicating that ethical leadership negatively moderates the job insecurity–job stress link by functioning as a buffering factor. It means that when the degree of ethical leadership is high, the increasing influence of job insecurity on job stress would be diminished, which supports Hypothesis 5 (Please See Figure 3).

### 4.4. Bootstrapping

The bootstrapping technique was implemented by using 10,000 samples [84] to test Hypothesis 4. The indirect mediation effect is significant at a 5% level if the 95% bias-corrected CI for the mean indirect mediation effect excludes zero [84]. Our results showed that the CI value for the mean indirect effect on the path (from job insecurity through job stress and organizational commitment to performance) excluded zero (95% CI = (−0.07, −0.01)). Therefore, the results indicate that the indirect sequential mediating influence is statistically significant with a level of 5%. This result validates our Hypothesis 4.

## 5. Discussion

### 5.1. Theoretical Implications

This research contributes to the job insecurity literature from three theoretical perspectives. First, by delving into the job insecurity–organizational performance link, we positively contribute to the flourishing body of job insecurity literature [17]. The previous research has underexplored the effects of job insecurity on organizational performance due to practical limitations, instead focusing on member attitudes, behaviors, physical/mental health, and individual level performance [17]. Although we acknowledge that those individual outcomes are important, organizational performance is one of the most crucial goals in the competitive business world. Also, the value of individual-level outcomes (e.g., attitudes, behaviors, physical, and mental health) should be evaluated in terms of organizational performance. Therefore, our attempt to examine how job insecurity influences organizational performance is meaningful.

Second, by investigating the underlying mechanisms in the relationship between job insecurity and performance, we help to resolve the inconclusive association between those variables in existing studies [17,19]. Some empirical studies have reported that job insecurity decreases individual-level performance [5,10], and other papers have found that job insecurity is not associated with performance [18,20,22]. On the other hand, some scholars have shown that job insecurity actually enhances the work-related motivations of employees [20,23]. Those inconclusive results could be due to a lack of empirical studies on the underlying mechanisms (i.e., mediators and moderators) within the link [17]. To resolve those discrepancies, we empirically tested and demonstrated that job insecurity negatively affects organizational performance by increasing employees’ job stress and thereby diminishing their organizational commitment. The results of this paper thus bolster previous works that suggested that job insecurity has a detrimental influence on performance.

Third, we revealed that ethical leadership functions as a critical buffering factor on the negative influence of job insecurity in an organization. Given that employees are likely to be affected by their leaders’ thoughts, words, and behaviors as they perceive what behaviors are appropriate or allowed in their organization [32,33], we expected leaders to play a critical role in employee interpretations of job insecurity. In other words, we showed that the increase in job stress caused by job insecurity can be diminished by a high level of ethical leadership. When the level of ethical leadership is high, employees perceive that they will be treated fairly and ethically, which allows them to understand and even accept their job instability. However, when employees feel that an unethical leader evaluates them unfairly, they are likely to perceive their job insecurity as unjustified, which can amplify their feeling of stress. Therefore, the ethical characteristics of a leader should be considered as a critical contingent factor in dealing with the negative effects of job insecurity.

### 5.2. Practical Implications

Our current research has practical implications for leaders and human resource management teams. First, our results suggest that the problem of job instability is critical to the competitive advantage of firms because it significantly diminishes the level of organizational performance. Our SEM demonstrates that job insecurity decreases organizational performance by increasing the level of employee job stress and decreasing employee commitment to the organization. Therefore, employee perceptions of job security are a crucial factor in predicting a fundamental organizational target—organizational performance. We thus propose that leaders and practitioners work to understand and deal with perceived job insecurity among employees. Leaders and practitioners have to effectively and wisely manage employee perceptions of job insecurity through various human resource management practices and systems.

Second, we propose that leaders and human resource management teams who want to diminish the perception of job insecurity develop efficacious indicators or indexes based on an accurate understanding of what factors are affected by which systems. By revealing that employee perceptions of job insecurity deteriorate organizational performance by increasing employee job stress and decreasing the level of organizational commitment, we have shown that leaders and human resource management teams should use the degree of job stress and organizational commitment to evaluate the effectiveness of their practices in addressing perceptions of job insecurity.

Third, we suggest that when leaders aim to reduce the harmful effects of job insecurity, they should understand and take advantage of the buffering effects of ethical leadership. To alleviate the negative effects of job insecurity on organizational performance, leaders have to behave ethically in their organization. Through training, top management teams can cultivate ethical leaders, which could help to diminish the negative influence that job instability can have in an organization.

### 5.3. Study Limitations and Future Works

Even though the current research has theoretical and practical implications, it still has important limitations. First, we could not sufficiently consider a variety of external features that influence job insecurity. We used member perceptions of job instability to measure the degree of job insecurity, which is a subjective scale. The scale we used has been well validated, and the concept of job insecurity is fundamentally a subjective perception. However, we could have and did not consider and incorporate the effects of “objective” features of job instability such as downsizing rates and HRM policies [11,12,16]. Future research should deal with this issue by adequately controlling for more factors.

Second, we could not use an objective scale to evaluate the degree of organizational performance. Instead, we used a subjective scale containing information from an HRM officer in each company. That subjective scale has its own academic and practical merits [11,12,16]—it has been validated [86] and shown to reflect objective organizational performance [57]. Nonetheless, future work on job insecurity should use objective performance scales such as sales and operating profits.

## Figures and Tables

**Table 1:** Demographic information.

Feature	Percent
**Gender**	
Men	47.2%
Women	52.8%
**Age (years)**	
20–29	22.6%
30–39	24.6%
40–49	26.6%
50–59	26.2%
**Education**	
Senior high school and below	13.6%
Community college	21.3%
Undergraduate	58.5%
Postgraduate and above	6.6%
**Job title**	
Staff	30.9%
Assistant manager	24.6%
Manager or deputy general manager	27.9%
Department/general manager or director and above	16.6%
**Occupation**	
Office workers	63.1%
Administrative positions	18.6%
Sales and marketing	6.7%
Manufacturing	5.3%
Education	2.0%
Other	4.3%
**Work experience**	
Less than 50 months	52.2%
50–100 months	20.2%
100–150 months	15.0%
150–200 months	5.3%
200–250 months	3.3%
More than 250 months	4.0%
**Firm size**	
Fewer than 50 employees	47.2%
50–99 employees	13.6%
100–299 employees	16.3%
300–499 employees	5.6%
More than 500 employees	17.3%
**Industry type**	
Manufacturing	25.9%
Services	14.6%
Construction	13.3%
Information services and telecommunications	10.7%
Education	9.3%
Health and welfare	7.3%
Public service and administration	7.3%
Financial/insurance company	3.3%
Other	8.3%

**Table 2:** Descriptive statistics.

	1	2	3	4	5	6	7	8	9
1. Job title_T2	-								
2. Work experience _T2	0.32 **	-							
3. Education_T2	0.12 *	−0.06	-						
4. Firm size_T2	−0.06	0.20 **	0.15 **	-					
5. Industry type_T2	0.03	0.01	0.06	−0.09	-				
6. Job Insecurity_T1	0.07	0.00	0.07	−0.04	−0.00	-			
7. Ethical Leadership_T1	0.04	0.05	−0.02	−0.00	0.04	−0.08	-		
8. Job Stress_T2	−0.03	−0.01	0.13 *	0.08	−0.08	0.24 **	−0.13 *	-	
9. Organizational commitment_T2	0.22 **	0.16 **	−0.02	−0.01	0.02	−0.09	0.44 **	0.37 **	
10. Organizational performance_T3	0.07	0.06	−0.07	−0.06	0.09	−0.03	0.39 **	−0.20 **	0.51 **

Note: * *p* < 0.05. ** *p* < 0.01. T1, T2, and T3 mean time point 1, 2, and 3.
